# Current Progress in the Rejuvenation of Aging Stem/Progenitor Cells for Improving the Therapeutic Effectiveness of Myocardial Repair

**DOI:** 10.1155/2018/9308301

**Published:** 2018-03-29

**Authors:** Gurleen Kaur, Chuanxi Cai

**Affiliations:** Center for Cardiovascular Sciences, Department of Molecular and Cellular Physiology, & Department of Medicine, Albany Medical College, Albany, NY 12208, USA

## Abstract

Ischemic heart disease affects a majority of people, especially elderly patients. Recent studies have utilized autologous adult stem/progenitor cells as a treatment option to heal cardiac tissue after myocardial infarction. However, donor cells from aging patients are more likely to be in a senescent stage. Rejuvenation is required to reverse the damage levied by aging and promote a youthful phenotype. This review aims to discuss current strategies that are effective in rejuvenating aging cardiac stem cells and represent novel therapeutic methods to treat the aging heart. Recent literature mainly focuses on three approaches that aim to reverse cardiac aging: genetic modification, pharmaceutical administration, and optimization of extracellular factors. *In vitro* genetic modification can be used to overexpress or knock down certain genes and allow for reversal of the aging phenotype. Pharmaceutical administration is another approach that allows for manipulation of signaling pathways related to cell proliferation and cell senescence. Since the stem cell niche can contribute to the age-related decline in stem cell function, rejuvenation strategies also include optimization of extracellular factors. Overall, improving the intrinsic properties of aging stem cells as well as the surrounding environment allows these cells to adopt a phenotype similar to their younger counterparts.

## 1. Introduction

Cardiovascular disease is the leading cause of mortality in the United States [1], and its risk increases in patients 65 years of age or older [2]. As the heart ages, the myocardium undergoes degeneration that leads to myocyte death [3]. Previous experiments conducted in the heart have explored whether the adult myocardium contains an undifferentiated pool of cells that may participate in cardiac repair [2]. The heart was initially thought to be a postmitotic organ without the capacity to replace itself. However, recent discoveries represent a major paradigm shift, suggesting that apoptotic cardiac cells are replaced by new cells derived from cardiac stem/progenitor cells (CPCs) [4]. Evidence has been obtained in favor of the regeneration of the aging myocardium. In a recent study, injection of autologous CPCs decreased scar size, increased the amount of visible myocardium, and improved regional function of the infarcted myocardium [5]. Local stimulation of CSCs can reverse the detrimental effects of aging on the heart and therefore represents a novel strategy for solving the problem of heart failure in the older population [2].

Obstacles to the success of stem cell-based clinical therapies include the poor survival of donor cells along with the age-related loss of stem cell regenerative capacity. More than 90% of transplanted mesenchymal stem cells (MSCs) die within the first few days [6]. Aging leads to diminished proliferative and differentiative potential due to increased oxidative stress, mitochondrial dysfunction, and genome instability [7]. Telomeres, repetitive nucleotide sequences at the ends of mammalian chromosomes that preserve chromosome stability and integrity, decrease in length with aging [8]. Accumulation of damage and shortening of telomeres leads to cellular senescence—a state of irreversible growth arrest [9]. Senescent cells are characterized by the incapability to contribute to tissue repair and regeneration. Aging is also associated with reactive oxygen species (ROS) that are generated by the mitochondria [3, 10, 11]. Mitochondrial overproduction of ROS also likely contributes to cellular senescence; it leads to the formation of highly reactive products O_2_ or H_2_O_2_, whose accumulation promotes senescence, DNA mutations, inflammation, and cell death pathways [8]. ROS can be detoxified within the cell by antioxidants such as superoxide dismutase (SOD) catalase, glutathione peroxidase, peroxiredoxin, and sulfiredoxin. However, an increase in ROS levels can subsequently alter the cell's normal redox state and provoke oxidative stress [12]. Therefore, rejuvenation is required to reverse the damage imposed by aging to restore tissue and organ function and improve longevity.

**Figure 1:**
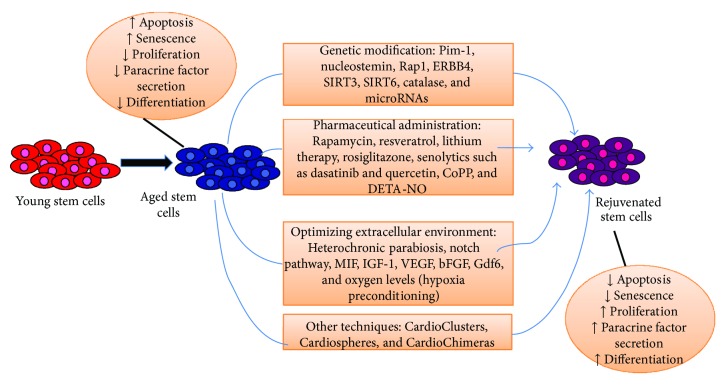
Summary of strategies used to rejuvenate aging stem cells and heal the injured heart. These methods result in an increase in proliferation and decrease in apoptosis and senescence, allowing for improved regeneration capabilities of the myocardium.

This review is designed to highlight current work in the field of rejuvenation of aging cardiac stem cells. Studies in this field have focused on three different approaches, summarized in Figure 1. The first category of strategies uses genetic modification to overexpress or knock down certain genes in cardiac stem cells. Certain proteins are found to either increase or decrease in expression in aging organisms, suggesting that reversal of this change in expression may rejuvenate older cells to a youthful phenotype. The second strategy for rejuvenation uses pharmaceutical administration to reverse senescence by targeting signaling pathways associated with important cellular processes such as proliferation, apoptosis, and senescence. Finally, the third strategy for rejuvenation involves optimizing the extracellular factors in order to prevent senescence and promote a youthful phenotype. A wide variety of stem/progenitor cells have been transplanted to improve cardiac regeneration, including skeletal myoblasts, hematopoietic stem cells, embryonic stem cells, and induced pluripotent stem cells [13–15]. However, this review focuses mainly on resident/adult mesenchymal stem cells and cardiac stem/progenitor cells.

## 2. Genetic Modification

*In vitro* genetic modification of aging cardiac stem cells to enhance proliferation, survival, and commitment is an effective strategy to enhance stem cell function and ensure improved cardiac output. Pim-1, a conserved serine/threonine protein kinase [1], is increased in expression in response to injury and protects against myocardial infarction [16] with its antiapoptotic and proproliferative actions [17]. Pim-1 kinase expression is higher in fetal human cardiac progenitor cells (hCPCs) as compared to older hCPCs, which suggests a correlation between Pim-1 expression and youthful phenotypic characteristic [18]. Recent studies have utilized hCPCs isolated from heart failure patients; hCPCs engineered to overexpress Pim-1 result in increased cellular engraftment and differentiation with improved vasculature [19]. Genetic modification with Pim-1 has the capability to rejuvenate with enhanced proliferation, decreased senescence, and increased telomere length [18]. A recent study found that Pim-1 expression in the heart coincides with nucleostemin (NS), a nucleolar protein required for cell cycle progression and proliferation. Reduced NS levels are associated with increased senescence and aging [20]. In addition, modification through Pim-1 is extremely powerful because of the recent study, in which Pim-1 targeted to the nucleus preferentially enhanced stem cell youthfulness associated with reduced senescence while Pim-1 targeted to mitochondria promoted increased interaction with antiapoptotic proteins such as Bcl-x [21]. These new findings suggest that organelle-specific overexpression of Pim1 may be utilized in a more personalized form of regenerative medicine based on the specific properties of the patient's hCPCs.

**Figure 2:**
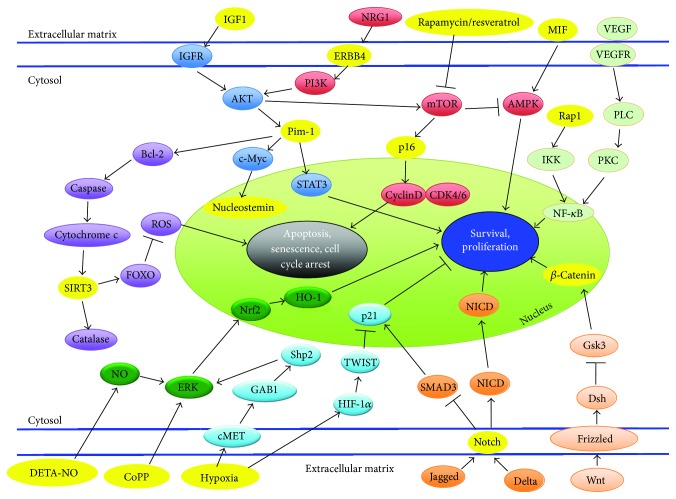
Molecular signaling pathways that are associated with rejuvenating aging stem/progenitor cells. Those that are currently being studied and were discussed in this review are highlighted in yellow.

Literature points to the paracrine effects of MSCs as being predominately responsible for cardiac repair. Some cytokines and growth factors produced by MSCs have been shown to be vital for cardiac protection while others are harmful for heart recovery. Therefore, optimization of MSCs before transplantation is required to maximize cell survival [22]. In a recent study, optimization was done through genetic modification of Rap1, a modulator involved in the NF-*κ*B pathway (Figure 2). Factors from the NF-*κ*B pathway have important roles in regulation of mitochondrial ROS, DNA replication, cell survival, and inflammation [23]. BM-MSCs with deletion of Rap1 were more tolerant than normal BM-MSCs to hypoxia that is associated with reduced activation of NF-*κ*B activity. Also, BM-MSCs with deletion of Rap1 showed a better therapeutic efficacy; they were associated with reduced inflammation postmyocardial infarction and enhanced cell survival of cardiomyocytes [24]. Another pathway that can be subjected to genetic modification and is also a paracrine regulator is the NRG1-ERBB4 signaling pathway (Figure 2). NRG1 is an essential paracrine regulator of cell-cell communication through activation of ERBB4, which further activates the PI3K/Akt pathway [25]. MSCs engineered with increased ERBB4 expression significantly preserve heart functions accompanied with reduced infarct size and enhanced cardiomyocyte division. A cardioprotective effect is seen due to activation of Akt and Bcl-2, which protects from apoptosis [26].

Aging is accompanied by a general decline in mitochondrial function in all tissues. Mitochondria may contribute to stem cell maintenance through regulation of specific metabolites such as NAD+ [27], whose effects are mediated by sirtuin family of NAD-dependent enzymes [28]. In myocardial tissue, SIRT3 localizes to mitochondria [29], reduces levels of reactive oxygen species (ROS), and blocks cardiac hypertrophic response through activation of Foxo-dependent antioxidants, manganese superoxide dismutase (MnSOD), and catalase [30]. Studies in mice revealed that SIRT3 expression was reduced in old aortic valves compared with young ones, signifying an age-associated SIRT3 reduction [31]. Overexpression of SIRT3 enhanced cells' ability to combat oxidative stress and reduced stress-mediated cell injury by activating catalase and MnSOD in human MSCs [32]. Another member of the sirtuin family, SIRT6, is a potential target for rejuvenation of aging stem cells [33]. A recent study demonstrated that knockdown of SIRT6 in human bone marrow MSCs resulted in impaired growth, proliferation, and migration ability, along with increased cell death and senescence [34]. Interestingly, recent results from our group showed that cytoglobin, a gene linked to oxidative stress and mitochondria respiration, promotes cardiac progenitor cell survival against oxidative stress via the upregulation of the NF*κ*B/iNOS signal pathway and nitric oxide production, providing a novel molecular target that can be used to enhance the effectiveness of cardiac stem/progenitor cell therapy for ischemic heart disease [35]. Overall, mitochondria-associated proteins represent a potential target for rejuvenation of aging cardiac stem cells. However, future *in vivo* studies need to be done in order to show improved therapeutic effectiveness in the heart.

miRNAs in the heart are also significant contributors to disease; their altered expression may be partly responsible for cardiovascular disorders [36]. Differential expression of miRNAs in old cells compared to young cells, such as downregulation of miR-17, miR-19b, miR-20a, and miR-106a in aged cells, implicates miRNAs as markers of biological aging [37]. Recent work has found an increase in expression of a specific miRNA, miR-195, which targets telomerase reverse transcriptase (Tert) gene, causing deterioration of the regenerative ability of old MSCs. Transplantation of old MSCs with knockdown of miR-195 led to improvement in cardiac function and reduction of infarct size [38]. Another study has reported that downregulation of miR-29c in MSCs led to the suppression of both the p53-p21 and the p18-pRB pathways and abrogation of cellular senescence [39]. Another miRNA, miR-34a, is elevated in mouse hearts after myocardial infarction [40]. Overexpression of miR-34a is associated with increased apoptosis, lower viability, and increased senescence, possibly through activation of SIRT1/FOXO3a pathway. Inhibition of miR-34a leads to fewer apoptotic cells and better viability [41] along with improved cardiac molecular signature and increased angiogenesis. Overall, miRNAs represent powerful therapeutic targets because they are small in length, around 22 nucleotides, and can be efficiently inhibited *in vivo* [42]. However, future work is needed to understand the mechanistic participation of various miRNAs in regulating cell senescence/aging.

Clinical trials, such as the SCIPIO phase 1, have supported the efficacy of CPC therapy [5]. However, ischemic heart disease is associated with increased age; therefore, it is necessary for cell-based therapies to reduce the harmful effects of aging. This can be done with genetic manipulation of the stem cells. Stem/progenitor cells can be extracted from a consenting patient through clinical methods and then isolated, cultured, and modified *in vitro* with genetic modification. The cells can be later transplanted back into the patient's own heart to assist in healing the damaged postinfarct myocardium [43]. Overall, Pim-1, nucleostemin, Rap1, ERBB4, SIRT3, SIRT6, and catalase along with various different miRNAs represent potential genes whose expression can be manipulated in order for stem cells to adopt a youthful phenotype. However, a limitation of genetic modification is that a combination of genes may need to be modified in order to effectively heal the aging infarcted heart. Overall, the fundamental goal in genetic modification is to eventually lead to an increase in proliferation and regenerative capacities of stem cells and inhibition of the senescent phenotype.

## 3. Pharmaceutical Administration

A variety of pathways (Figure 2) including the mTOR/PI3K, WNT/*β*-catenin, ERK/NRF2, and STAT3/NF*κ*B can be targeted for rejuvenation by pharmacological manipulation. Drugs such as rapamycin can be used to rejuvenate aging cardiac stem cells. Rapamycin is an inhibitor of mammalian target of rapamycin (mTOR) [44], a major downstream component in the PI3K senescence pathway [45]. mTOR's inactivation by rapamycin inhibits the TORC1 complex [46] and brings cells from a senescent to a quiescent stage [47]. A recent study utilized rapamycin along with resveratrol, a drug known for activating AMPK, which increases mitochondrial biogenesis and function [48]. The combination of these drugs was found to modify the secretome of cardiac stem cells from explanted decompensated hearts (E-CSC) such that there was prevention of cardiomyocyte death and senescence. Additional investigations in which infarcted mice were injected with E-CSC treated with rapamycin and resveratrol showed improved cardiac output. Using drugs such as rapamycin and resveratrol has a high potential since it avoids the possible detrimental effects from genetic modification [49]. As with any drug, rapamycin has side effects, and future work is needed to expose whether side effects could limit its usage [44].

Another research group has identified the wingless integration (WNT)/*β*-catenin pathway (Figure 2) as a potential target for rejuvenation of hMSCs used in stem cell therapy for cardiac repair [9]. The study showed that increased age was associated with reduced MSC proliferation, MSC differentiation, and WNT/*β*-catenin signaling. However, some functions of MSCs from aging individuals could be revitalized with lithium therapy, which increases *β*-catenin availability to improve myogenic differentiation [50]. The WNT/*β*-catenin pathway is closely related to stem cell renewal and differentiation through regulation of CTNNB1, which plays an essential role in cardiogenic development [9].

Targeting senescent cells is another possibility that can lead to rejuvenation through pharmaceutical administration. Most senescent cells express p16^Ink4a^, a cyclin-dependent kinase inhibitor that leads to cell arrest by activating Rb. The expression of p16^Ink4a^ is also known to increase with aging (Figure 2). A novel transgene, INK-ATTAC, was used to eliminate p16^Ink4a^-positive senescent cells upon administration of rosiglitazone to induce senescence. The administration and resulting clearance of senescent cells led to enhanced health span and a delay in multiple age-related phenotypes in progeroid mice [51]. Furthermore, senolytics, a new class of drugs that selectively kill senescent cells, represent a great potential for improving health span. Two drugs, dasatinib (D) and quercetin (Q), were successful in improving cardiac functioning in aging mice and reducing the number of senescent bone marrow-derived murine MSCs by interfering with the antiapoptotic and prosurvival mechanisms of senescent cells. The results indicate that senescent cells exert deleterious effects on cardiovascular function with aging, and that clearance of these cells represents a novel therapeutic approach for rejuvenation [52].

Pharmacological treatment with cobalt protoporphyrin (CoPP) is another method to improve therapeutic effectiveness of myocardial repair. CoPP is an inducer of heme oxygenase-1 (HO-1), which induces cellular protection. A previous study has shown that preconditioning of hCSCs with CoPP increases the resistance of these cells to oxidative stress-induced apoptosis via the activation of ERK/NRF2 signal pathways (Figure 2). Knockdown of HO-1 leads to a diminished cytokine effect, which suggests that the beneficial effects of CoPP preconditioning may be due to the secretion of protective cytokines [53]. Another study demonstrated that CoPP treatment in hypoxic cells reduced cell damage and increased the viability of cardiomyocytes by preserving mitochondrial membrane potential [54]. Furthermore, preconditioning hCSCs with CoPP leads to an increase in cell survival and proliferation after transplantation into the infarcted heart along with greater LV functional and structural improvement [55]. Overall, the HO-1 inducer, CoPP, is a promising candidate that can improve the efficiency of CSC-based therapies for ischemic heart disease. Nitric oxide (NO) is a gaseous signaling molecule shown to have an antiapoptotic role in many cells. Diethylenetriamine nitric oxide adduct (DETA-NO) is a chemical-based NO releaser. A recent study has demonstrated that preconditioning human cardiac stem cells with DETA-NO promotes cell survival and resistance to oxidative stress. Future work would require examination of the *in vivo* survival of hCSCs enhanced by DETA-NO preconditioning [56].

Pharmaceutical administration is a valid methodology for rejuvenation. Similar to the genetic modification technique, stem/progenitor cells can be extracted from a patient and cultured *in vitro* while being treated with pharmaceutical agents. However, a potential limitation of this strategy is that pharmaceutical agents can have a wide variety of effects, and therefore, a balance between side effects and benefits must be reached before the drugs can be used in a clinical setting. Perhaps, a combination of pharmacological treatment and genetic modification can be employed to rejuvenate aging stem cells, allowing for a more comprehensive approach.

## 4. Optimization of Extracellular Factors

The host environment plays a very important role in the outcome of cell therapy; allogenic cells from young, healthy donors have been used to overcome age-related stem cell dysfunction [57]. Furthermore, tissue aging is influenced by systemic and circulating factors. The detection of senescence factors by neighboring healthy cells might further progress cellular senescence, contributing to dysfunction associated with age-related cardiac diseases [2]. Factors that slow the age-dependent deterioration of the cell niche represent a new method for treating age-related diseases [58]. Using systemic and circulating factors to rejuvenate aging cells has been shown in nerve [58] and bone [59] cells. Heterochronic parabiosis experiments, in which a young and old mice are surgically linked so they develop a shared blood circulation [60], indicate that signals from a young circulation can impact the function of aging tissues [61].

Manipulation of the Notch pathway, in which ligands such as Delta interact with the Notch receptor (Figure 2), can be used to rejuvenate aging cardiac stem cells [62]. Studies have found that the failure of this pathway to be activated can lead to decline in the regenerative potential of muscle with age, due in part to impairment in upregulation of the Notch ligand Delta after muscle injury [63]. Using heterochronic parabiosis to restore Delta upregulation in aging satellite cells allows for enhanced activation and proliferation [64]. Notch activation has been found to restrain cardiac hypertrophy and fibrosis and promote cardiac precursor expansion. Communication between Jagged1-expressing cardiomyocytes and Notch-expressing MSCs is important to shift the response towards cardiac precursor expansion [65]. Activation of Notch signaling in the border zone after infarction promotes survival and improves cardiac function. Delivery of a peptide mimic of the Notch1 ligand Jagged1 to the infarcted rat heart led to improvements in cardiac function and contractility [66]. Notch signaling also plays a crucial role in cell senescence; overexpression of Notch prolongs the lifespan of vascular endothelial cells by inhibiting a p16-dependent pathway [62]. Overall, manipulation of the Notch signaling pathway could be a new therapeutic target for treating age-associated vascular diseases.

Another circulating factor implicated in cardiac disease is insulin-like growth factor 1 (IGF-1). IGF-1 is important in the recovery process of the heart because of its subsequent activation of PI3K/Akt signaling (Figure 2), which allows for enhanced cell survival, release of growth factors, stem cell mobilization, and angiogenesis. IGF-1 overexpression also accentuates the release of various growth factors, including HGF and VEGF, which contribute towards reduced cell apoptosis. Localized IGF-1 overexpression also significantly preserves LV wall thickness and contractile function *in vivo* [67]. A study has concluded that the IGF-1 receptor (IGF-1R) identifies a pool of human cardiac stem cells that have a superior therapeutic potential for myocardial regeneration. Overall, the presence of IGF-1R led to decreased apoptosis rates, enhanced telomerase activity, preserved telomere integrity, and favoring of hCSC division and survival. The expression of IGF-1R and the synthesis of IGF-1 are attenuated in aging hCSCs. Therefore, the study shows that a careful analysis of the phenotypic properties of the cells can be used to consider which cells are used for clinical treatment [68].

Many other cytokines and growth factors have been implicated as potential candidates for rejuvenation. Macrophage migration inhibitory factor (MIF) (Figure 2) is a cytokine that is released by ischemic cardiomyocytes in the heart, allowing for protection from injury and cellular apoptosis [69]. MIF-treated aged MSCs survived better than young MSCs not treated with MIF, suggesting that MIF possesses antiapoptotic properties. MIF also restored the trophic activity of MSCs as seen by the quantification of VEGF, HGF, and IGF levels [70]. Furthermore, tissue engineering has been used to seed angiogenic cytokines, VEGF and bFGF, onto a collagen scaffold. The cytokine-enhanced, tissue-engineered patch rejuvenated aging cells, prolonged cell survival, and improved angiogenesis to restore ventricular function [71]. Gdf6, found to be downregulated in old MSCs, is another growth factor that may play a role in rejuvenation. A recent study in which Gdf6 was administered led to the restoration of the differentiation potential of aged MSCs *in vitro* and wielded beneficial effects on age-associated pathologies in mice [72]. An understanding of the mechanisms underlying the regenerative effects of various growth factors could lead to the development of novel therapeutic agents for the treatment of myocardial infarction.

Oxygen levels of stem cell niches are known to play a role in stem cell quiescence; changing oxygen concentrations can affect the survival and proliferation of stem cells when used for myocardial therapy. It has been shown that expansion of hCSCs under hypoxic conditions leads to greater engraftment and functional benefit after implantation into infarcted hearts of mice [73]. Further studies show that MSCs cultured in hypoxia activate the Akt and HGF-cMet signaling pathway (Figure 2), which leads to increase in migration rates. The culture in hypoxic conditions is also beneficial because it is more similar to the physiologic niche of MSCs in the bone marrow. A hind limb ischemia injury model was used to show that mice that received hypoxic preconditioned MSCs recovered faster than control groups [74]. A study with EPCs showed that hypoxia can prevent senescence, increase proliferation capacity and lifespan, and maintain the stem cell properties of EPCs through HIF1*α*-induced TWIST expression (Figure 2), which inhibits cell cycle arrest and replicative senescence [75]. Another study displayed a significant increase in the expression of prosurvival proteins such as NF-*κ*B and antiapoptotic proteins Bcl-2 and Blc-xL. Hypoxia-preconditioned MSC transplantation leads to enhanced angiogenesis and vascularization as compared to normal MSCs. Since 90% of grafted cells die within the first few days of transplantation, this protective effect of hypoxia is crucial to protect transplanted cells [6].

The identification of rejuvenating growth factors and environmental conditions opens new prospects to reverse the effects of cardiac aging. This strategy allows for preconditioning of the stem cells in order to optimize the extracellular environment before transplantation into the postinfarct heart. Altering systemic expression of factors that promote stem cell activity and culturing cells in hypoxic conditions are powerful methods of rejuvenation. However, the stem cell niche is very complex; therefore, further research is required before this strategy can lead to clinical translation.

## 5. Other Strategies

Other engineering strategies are being developed to rejuvenate the senescent heart. Two novel stem cell products have been engineered: CardioChimeras and CardioClusters. CardioChimeras are formed by fusing together CPCs with MSCs. Combined administration of hMSCs and human c-kit-positive CPCs into an infarcted heart has significantly improved myocardial structure and function [76]. This technique creates a cell hybrid that combines optimal traits such as proliferation, survival, and paracrine secretion. In addition, CardioClusters are comprised of a 3D microenvironment consisting of specific cell types isolated from the human heart such as CPCs, MSCs, EPCs, and fibroblasts. Furthermore, Cardiospheres are 3D spontaneous aggregations of heterogeneous stem cells and have been created to enhance communication between stem cells and the cardiac environment [9]. A clinical trial CADUCEUS has explored the effect of intracoronary infusion of cardiosphere-derived cells and found a significant reduction in scar mass [77]. CardioClusters, Cardiospheres, and CardioChimeras represent a next generation of stem cell therapy that can be used to rejuvenate the heart. However, these combinatorial cell therapies are limited because of differences in proliferation rates and survival after injection into the damaged heart [9]. More studies are required before this technique can be translated into clinical applications.

## 6. Conclusion and Future Directions

This review discusses the current strategies for the rejuvenation of aging stem cells in order to improve therapeutic techniques for myocardial repair. Cardiac stem cells have an innate ability to rejuvenate the myocardium; however, the aging population of patients has a compromised stem cell population in terms of functional capacity and regenerative potential [78]. Therefore, these aging stem cells must be rejuvenated by techniques such as the discussed methods of *in vitro* genetic modification, pharmaceutical administration, and optimization of extracellular factors. Recent studies show promising results of the ability of these techniques to rejuvenate the aging heart. However, more understanding of the combinatorial effects of these interventions and fine-tuning of these techniques, depicted in Figure 1, is required to evaluate the translational potential of these methods. Each strategy has its own advantages and disadvantages as outlined in Table 1. Overall, the success of myocardial regenerative treatment will require teamwork across various disciplines to make stem cell therapy a reliable method for cardiac repair.

## Figures and Tables

**Table 1:** Advantages and disadvantages of the various strategies that were discussed.

	Genetic modification	Pharmaceutical administration	Optimization of extracellular factors
Advantages	(i) Has stable effect (ii) Can directly target specific cell survival pathways	(i) Has the ability to effect multiple pathways at once (ii) Can lead to release of growth factors and cytokines	(i) Takes into account the stem cell niche (ii) Has potential to prime cells to endure the postinfarct environment
Disadvantages	(i) May need multiple genes modified to have a significant effect	(i) Has potential side effects	(i) Has multiple aspects of environment may need to be optimized to have significant effect
